# Approaches of integrating the development of guidelines and quality indicators: a systematic review

**DOI:** 10.1186/s12913-020-05665-w

**Published:** 2020-09-16

**Authors:** Miranda W. Langendam, Thomas Piggott, Monika Nothacker, Arnav Agarwal, David Armstrong, Tejan Baldeh, Jeffrey Braithwaite, Carolina Castro Martins, Andrea Darzi, Itziar Etxeandia, Ivan Florez, Jan Hoving, Samer G. Karam, Thomas Kötter, Joerg J. Meerpohl, Reem A. Mustafa, Giovanna E. U. Muti-Schünemann, Philip J. van der Wees, Markus Follmann, Holger J. Schünemann

**Affiliations:** 1 Department of Clinical Epidemiology, Biostatistics and Bioinformatics, Amsterdam UMC, University of Amsterdam, Amsterdam Public Health institute, Amsterdam, Netherlands; 2 Department of Health Research Methods, Evidence, and Impact, McMaster University, Hamilton, Canada; 3 Institute of Medical Knowledge Management, Association of the Scientific Medical Societies, Berlin, Germany; 4 Department of Medicine, University of Toronto, Toronto, ON Canada; 5 Farncombe Family Digestive Health Research Institute, McMaster University, Hamilton, Canada; 6 Australian Institute of Health Innovation, Macquarie University, Level 6, 75 Talavera Rd, Sydney, Australia; 7 Department of Pediatric Dentistry, Dental School, Federal University of Minas Gerais, Belo Horizonte, Brazil; 8 IKOetxe – Ikerkuntza Osaungintza, Health Research, Gipuzkoa, Irun, Basque Country Spain; 9 Department of Pediatrics, University of Antioquia, Medellin, Colombia; 10 Coronel Institute of Occupational Health and Research Center for Insurance Medicine, Amsterdam UMC, University of Amsterdam, Amsterdam Public Health Research Institute, Amsterdam, The Netherlands; 11 Institute of Family Medicine, University Medical Center Schleswig-Holstein, Campus Lübeck, Lübeck, Germany; 12 Institute for Evidence in Medicine, Medical Center and Faculty of Medicine, University of Freiburg, Freiburg, Germany; 13 Department of Internal Medicine, University of Kansas Medical Center, Kansas, USA; 14 Department of Systemic Pathology, Vita Salute San Raffaele University, Milan, Italy; 15 Department of Rehabilitation and IQ healthcare, Radboud Institute for Health Sciences, Radboud University Medical Center, Nijmegen, Netherlands; 16 German Cancer Society, Freiburg, Germany; 17 Department of Medicine, Hamilton, McMaster University, Hamilton, Canada; 18 Department of Health Research Methods, Evidence and Impact, McMaster University Health Sciences Centre, Room 2C16, 1280 Main Street West, Hamilton, ON L8N 4K1 Canada

**Keywords:** Guidelines, Recommendations, Quality improvement, Quality assurance

## Abstract

**Background:**

Guidelines and quality indicators (for example as part of a quality assurance scheme) aim to improve health care delivery and health outcomes. Ideally, the development of quality indicators should be grounded in evidence-based, trustworthy guideline recommendations. However, anecdotally, guidelines and quality assurance schemes are developed independently, by different groups of experts who employ different methodologies. We conducted an extension and update of a previous systematic review to identify, describe and evaluate approaches to the integrated development of guidelines and related quality indicators.

**Methods:**

On May 24th, 2019 we searched in Medline, Embase and CINAHL and included studies if they reported a methodological approach to guideline-based quality indicator development and were published in English, French, or German.

Results: Out of 16,034 identified records, we included 17 articles that described a method to integrate guideline recommendations development and quality indicator development. Added to the 13 method articles from original systematic review we included a total 30 method articles. We did not find any evaluation studies. In most approaches, guidelines were a source of evidence to inform the quality indicator development. The criteria to select recommendations (e.g. level of evidence or strength of the recommendation) and to generate, select and assess quality indicators varied widely. We found methodological approaches that linked guidelines and quality indicator development explicitly, however none of the articles reported a conceptual framework that fully integrated quality indicator development into the guideline process or where quality indicator development was part of the question formulation for developing the guideline recommendations.

**Conclusions:**

In our systematic review we found approaches which explicitly linked guidelines with quality indicator development, nevertheless none of the articles reported a comprehensive and well-defined conceptual framework which integrated quality indicator development fully into the guideline development process.

## Contributions to the literature

- Quality indicators are used to monitor guideline adherence as they measure structures, processes and health outcomes of care.
- Ideally, development of the quality indicators should be integrated in the guideline development process to establish a direct link with the recommendations.
- We extended and updated a systematic review on existing approaches for integrated development and found that quality indicators development is a topic of high interest, but there is minimal methodological advancement and the connection with guideline development methods is very limited.
- A well-defined methodological framework to integrate quality indicator development fully into the guideline development process is needed.

## Introduction

Guidelines and quality assurance (QA) schemes both aim to improve health care delivery and health outcomes. A QA scheme is a common set of quality and safety requirements for health care service. It covers interventions and services and may include several quality dimensions. Quality indicators are used to benchmark the fulfilment of a requirement using a clearly defined numerator and denominator (ISO 9000:2015 Quality management systems - Fundamentals and vocabulary (www.iso.org/standard/45481.html). Quality indicators are measurable items referring to structures, processes, and outcomes of care [1]. Ideally, the development of quality indicators should be grounded in evidence-based health care recommendations, derived from trustworthy guidelines.

However, anecdotally, guidelines and quality assurance schemes are developed separately, in isolation, by different groups of experts who employ different methodologies. It is often unclear, for example, how QA organizations (e.g. International Society for Quality in Healthcare) and guideline developers (e.g. the World Health Organization or professional societies) interact and how, when and in which context guideline recommendations are used to develop QA schemes or quality indicators. This lack of coherence may have important adverse consequences for implementation and adherence with respect to guidelines and QA schemes.

There is potential benefit of aligning activities and methods, resulting in an integrated approach. In this context integration means that QA scheme or quality indicator development is considered in all steps of guideline recommendation development, starting with the formulation of the key questions and defining the outcomes. Integration will then result in a set of quality indicators (and QA scheme) that is directly related to the key questions and recommendations in the guideline. The European Commission (EC) in its European Commission Initiative on Colorectal Cancer (ECICC) is exploring ways to integrate guideline recommendation and QI development.

To inform the ECICC, we performed a systematic review in order to identify and evaluate the current approaches to guideline-based quality indicator development, which is presented in this paper. We also performed a feasibility study, creating a proposal for uniform definitions of QI, performance measures and performance indicators, and we organized a three-day expert workshop, the outcomes of which are published elsewhere (refs Terminology and TwoWorlds paper).

The objectives of this systematic review were twofold. First, to identify and describe approaches that are utilized to develop guideline recommendations and quality indicators, i.e. in an integrated framework. Second, to evaluate the effects of an integrated guideline and quality indicator development approach on individual health outcomes as well as process and structure outcomes (e.g. time required to develop recommendations and quality indicator, feasibility, acceptability by key stakeholders, and development costs).

## Methods

We initially performed a systematic review of peer-reviewed and grey literature to identify approaches in which guideline recommendations and QI are developed in an integrated framework. The protocol with detailed methods description is published in the Prospective Register of Systematic Reviews (PROSPERO, CRD42018097302). We then identified a published systematic review on this topic that was current until April 2010 [2]. We contacted the lead author who agreed to collaborate on an update of that review by first applying the original search strategy and eligibility criteria and expanding it by searching for additional articles and reports.

### Data sources and searches

The eligibility criteria of the original review were English, French, or German articles reporting at least one methodological approach to guideline-based quality indicator development. We searched in Medline, Embase and CINAHL. All study and publication types were included. Studies at the full-text screening stage that did not describe the extraction of recommendations from clinical guidelines in detail were excluded [2].

We refined the eligibility criteria. Reports describing or evaluating approaches in which QA schemes or quality indicators are developed simultaneously or integrated with health-related evidence-based guideline recommendations were eligible for inclusion. Development of guideline recommendations should be based on evidence summaries, while quality indicator development could be based on evidence or expert consensus, or a combination of the two.

We updated the search as described by the original review by Kötter et al., from 2010 to May 24th, 2019 (See Additional file 1: Appendix A for details on search strategy). In addition, we actively searched for manuals or methods articles that apply guideline-based methods but do not describe that method in detail (topic articles) and we expanded the list of institutional websites (Additional file 1: Appendix B).

### Study selection

Two reviewers independently selected potentially eligible articles by screening titles and abstracts followed by full-text screening (Additional file 1: Appendix C). Disagreements were resolved by discussion, or with the help of a third reviewer.

### Data extraction and quality assessment

Characteristics of the approaches meeting inclusion criteria were abstracted into a standardized form (Additional file 1: Appendix D) [2]. Data were extracted by one reviewer and checked for accuracy by a second reviewer.

For evaluation studies we planned to evaluate the risk of bias of the included studies with a tool appropriate for the study design. However, we did not find any of these studies.

### Data synthesis and analysis

We structured the data in two ways. First, we matched the reported quality indicator process to the guideline development process using the Guidelines International Network (GIN)-McMaster Guideline Checklist (which currently does not include a quality indicator section) [3]. Second, we described the results using the items of the GIN Reporting standards for guideline-based performance measures of GIN [4].

## Results

### Search and selection

**Fig. 1:**
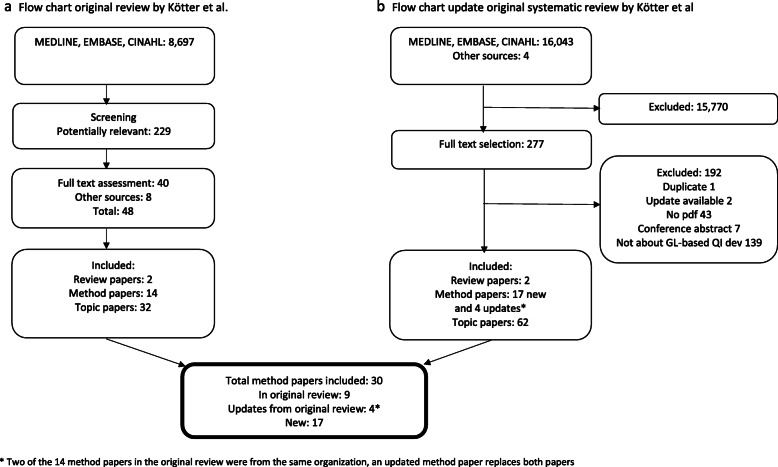
**a** Flow chart original review by Kötter et al. **b** Flow chart update original systematic review by Kötter et al.

Figure 1 presents the flowcharts with the results of the search and selection. The review by Kötter et al. included 48 articles [2]. Of these, 14 articles were method articles, 32 articles were topic articles and 2 were review articles.

In the update (May 24th, 2019) the total number of references from the electronic databases was 16,034 records, of which 273 were screened as potentially relevant. We found an additional 4 articles via other sources (via experts). Of these, 139 articles were not about guideline-based quality indicator development, 7 were conference abstracts, for 43 we could not retrieve the full text, for 2 articles an update was available and 1 article was a duplicate of another – these 192 articles were excluded. The remaining 85 articles were included: 17 new method articles, 62 topic articles and 2 review articles. Four articles were updates from articles in the original review [5–8]. Two of the 14 method papers in the original review were from the same organization, an updated method paper replaces both papers. Except for the articles with an updated version there was no overlap in included studies between the original review and the update. Thus, in total, 30 method articles were included of which 17 were not included in the prior review [9–25].

The review of institutional websites did not reveal any additional method articles. We did not identify additional method articles among the topic articles and there were no evaluation studies.

### Characteristics of the included articles

Table 1 presents the study characteristics. The articles were authored by a wide variety of professional societies, universities and governmental organizations across different healthcare settings and clinical topics, based in the United States (*n* = 11), United Kingdom (*n* = 6), Netherlands (*n* = 4), Germany (*n* = 3), Canada (*n* = 2), Belgium (*n* = 1) and Japan (*n* = 1)). Two articles were authored by an international group.

**Table 1:** General characteristics of the included method papers

Reference	Institution (country)	Topic	Setting	Funding
Advani 2003 [26]	BMIR (US)	Hypertension	–	Public
AHCPR 1995 [27] AHRQ 1995 [28] Hughes 2008 [8]	AHRQ (US)	–	–	Combined public/private
AQUA 2010 [29] AQUA 2013 [7]	AQUA (DE)	–	–	Not reported
ÄZQ 2011 [30] Nothacker 2011 [6]	ÄZQ (DE)	Chronic heart failure	–	Public/private
Baker 1995 [31]	Eli Lilly National Clinical Audit Centre (UK)	–	–	Not reported
Bayley 2018 [15]	Institute National d‘Excellence en sante et en services sociaux (INESS/ONF) (CA)	Traumatic brain injury	–	Public
Califf 2002 [32]	DCRI (US)	Cardiovascular medicine	All	Public
Cottrell 2018 [24]	American Rhinological Society (US)	Chronic rhinosinusitus	All	Not reported
Cheng 2010 [9]	American Academy of Neurology (US)	Parkinson disease	All	Not reported
Davies 2011 [19]	University Bristol (UK)	Wound, end of life and diabetes care	Outpatient care	Public
Duffy 2005 [33]	APIRE (US)	Bipolar disorder	Outpatient care	Not reported
Fiset 2019 [23]	University of Ottawa School of Nursing (CA)	Pain management	Inpatient, outpatient, long-term care, palliative	Not reported
Follmann 2017 [14]	German Guideline Program in Oncology (GGPO) (DE)	Oncology		Not reported
Golden 2008 [34]	UAMS (US)	Bipolar disorder	Rehabilitation	Public
Graham 2009 [35]	Immpact (UK)	–	All	Public
Hommel 2016 [16]	Internal Medicine and IQ healthcare, Radboud UMC (NL)	Perioperative diabetes care	All	Public
Hutchinson 2003 [36]	ScHARR (UK)	CHD	Hospital	Combinedpublic/private
Kahn 2014 [11]	American Thoracic Society (ATS) (US)	Pulmonary, critical care, and sleep medicine	–	Not reported
Laclair 2001 [37]	VA Medical Center (US)	Stroke	Rehabilitation	Public
Luitjes 2013 [25]	Dutch Society for Obstetrics and Gynaecology and IQ healthcare, Radboud UMC (NL)	Hypertensive diseases in pregnancy	Hospital	Public
Mazzone 2014 [10]	American College of Chest Physicians (CHEST)(US)	Lung cancer	District nursing	Private non-profit
Rushforth 2015 [20]	University Leeds (UK)	–	Hospital	Public
Schleedoorn 2016 [13]	EndoKey Group (international)	Endometriosis	Hospital	No funding
Spertus 2005 [38] Spertus 2010 [5]	American Heart Association (US)	–	Hospital	Public
Sutcliffe 2012 [18]	NICE (UK)	–	Primary care	Public
Ten Berg 2019 [21]	Dutch Childhood Oncology Group	Pediatric febrile neutropenia	Hospital	Public
Ueda 2019 [22]	Kyoto University, Nara Medical University (Japan)	Low-risk labour care	Hospitals	Public
Vasse 2012 [17]	International	Psychosocial care in dementia	All	Public
Werbrouck 2013 [12]	EFFECT project/KCE (BE)	Uterine cancer	Hospital	Private non-profit
Wollersheim 2007 [39]	IQ healthcare, Radboud UMC (NL)	Oncology, diabetes, antibiotics for pneumonia	–	Not reported

### Approaches that link guideline recommendations and QI development

For a detailed overview we organised the results of the 30 method articles in two ways. The first way is the GIN-McMaster Guideline checklist (Table 2), to match the domains in the guideline process with the accompanying domain in QI development [3]. The second way is the GIN Reporting standards for guideline-based performance measures. This reporting standard includes 9 items of the quality indicator development process [4]. We used these items to provide a detailed overview of the 30 method articles (Table 3).

**Table 2:** Methods of guideline-based QI development matched to the steps in the guideline development process

Item on GIN-McMaster Guideline checklist	Results in methods papers on QI development
1. Organization, Budget, Planning and Training	Funding: 14 of the 30 approaches were publicly funded, 3 were privately funded, 2 were funded both publicly and privately, 1 did not receive funding and for 10 funding was not reported.
2. Priority Setting	See 5.
3. Guideline Group Membership	Criteria for selection of GDG members were reported in 6 articles. Four articles reported selection of a multidisciplinary panel, including methodological competence, experience in quality improvement, policy decision making and knowledge translation. All 6 articles mentioned clinical expertise for the specific health care topic as competence. Criteria for selection of QI development panel members were mentioned in 15 articles. Clinical expertise was a criterion in all 15 articles, methodological experience was reported in 6 of the 15 articles. Patients/lay persons were part of three panels. Six reports did not use a formal panel and in 9 articles the criteria were unclear.
4. Establishing Guideline Group Processes	Group processes were not described in any article.
5. Identifying Target Audience and Topic Selection	Fifteen articles reported criteria for selecting the QI topics and the target audience. The criteria, and phrasing of the criteria, varied from article to article. Criteria for topic selection included relevance for the specific care domain (e.g. primary care), quality of care gap, sound evidence base, feasibility, availability, measurability, reliability, validity, regulatory requirements, unknown quality adherence, expected impact on quality of life, costs, work load, disease severity, potential to reduce health inequities and covering all aspects of the care process.
6. Consumer and Stakeholder Involvement	Patients were included in the QI selection process in 9 of the 30 articles.
7. Conflict of Interest (COI) Considerations	Conflicts of interest considerations for the QI development process were not mentioned in any of the papers.
8. (PICO) Question Generation	See item 5 (QI topic selection).
9. Considering Importance of Outcomes and Interventions, Values, Preferences and Utilities	Seventeen articles reported criteria for QI selection. In 9 of these articles patient outcomes, health gain or importance or clinical effectiveness were part of the criteria.
10. Deciding what Evidence to Include and Searching for Evidence	See item 11.
11. Summarizing Evidence and Considering Additional Information	All articles used evidence of guidelines as starting point for the QI development (this was an inclusion criterion). Thirteen articles report additional sources, e.g. literature searches for existing QI sets or available data. In 7 articles QI development was based on multiple guidelines, and in 9 articles QI were developed based on one guideline. In 1 article this was not specified.
12. Judging Quality, Strength or Certainty of a Body of Evidence	8 of the 13 articles which report criteria for selecting recommendations as basis for QI development use level of evidence as a criterion; 3 of the 8 approaches use GRADE and suggest that only strong recommendations should be considered for translation into QI.
13. Developing Recommendations and Determining their Strength	See item 12.
14. Wording of Recommendations and of Considerations of Implementation, Feasibility and Equity	Feasibility was mentioned as a criterion for selecting QI (10 articles). Equity was mentioned once, as a criterion for selecting the topic for which the QI were developed.
15. Reporting and Peer Review	Reporting and peer review of QI were not mentioned.
16. Dissemination and Implementation	Implementation: 12 articles report a QI implementation plan as part of their approach, mostly consisting of development of tools and software, and audits.
18. Updating	Updating of QI was not explicitly mentioned in any of the papers.

**Table 3:** Guideline-based QI development reporting standard items and report of these criteria in the method papers

Reporting standard item	Reported in method papers
1a. Guideline selection: criteria	Selection of guidelines was based on topic and • evidence-based development (*n* = 18) • methodological quality of the guideline (*n* = 2) • use of GRADE (*n* = 1) • structured format (*n* = 1) • no other criterion (*n* = 1) • unclear (*n* = 7)
1b. Guideline selection: appraisal of guidelines	• AGREE (*n* = 8) • criteria not fully specified (*n* = 4) • not reported (*n* = 18)
2. Selection of guideline recommendations	• based on topic (*n* = 2) • impact on patient outcome (n = 4) / burden of illness (n = 1) / clinical utility (*n* = 1) / available treatment (*n* = 1) • relevance (*n* = 4) / appropriateness (*n* = 1) • value for money (*n* = 1) • practice variability (*n* = 1) • scope for improvement (*n* = 1) / gap in quality of care (*n* = 1) • priority / feasibility for implementation (*n* = 3) • validity (*n* = 2) / reliable (*n* = 1) • (high) level of evidence (n = 8) / adequate scientific proof (*n* = 1) • direct link to aim of guideline (*n* = 1) • common to more than one guideline (*n* = 1) • unclear (*n* = 3) • no selection (*n* = 6)^a^
3. Selection of performance measures from recommendations	• formal panel method (*n* = 11) • other or informal consensus method (*n* = 13) • not reported (n = 2) • unclear (n = 4)
4. Core attributes of performance measures (criteria for selecting QI)	• relevance (n = 4) • potential for improvement (*n* = 9) / likely to change current practice (n = 2) / gap in quality of care (*n* = 2) / importance for health care (*n* = 4) • burden of illness (*n* = 2) / improving patient outcomes (*n* = 9) • cost-effectiveness (*n* = 4) • influenced by service provider (n = 3) • appropriateness (*n* = 1) • evidence base/scientific soundness (*n* = 7) • (strength of) association with patient important outcome (*n* = 2) • feasibility (*n* = 7) • no risk for unintended consequences (*n* = 3) • unambiguous definition (*n* = 2) / clear (*n* = 1) • data routinely collected (*n* = 1) • measurable (*n* = 4) / interpretable (*n* = 1) / actionable (*n* = 2) • applicable (*n* = 3) / acceptable (*n* = 1) / adherence (*n* = 1) • reliable (*n* = 6) / face validity (*n* = 2) / construct validity (*n* = 1) / content validity (*n* = 2) • precision (*n* = 1) • minimum bias (*n* = 1) • not reported (*n* = 4) • unclear (*n* = 3)^a^
5. Specification of performance measures Numerator and denominator is specified unambiguously and in detail.	• denominator: population eligible to receive the clinical interventions, numerator: desired intervention and subset of population that should receive it (*n* = 6) • based on algorithm (*n* = 1) • formulation of numerator and denominator in line with formulation of recommendation (*n* = 1) • numerator and denominator including risk adjustment factors (*n* = 3) • clinical researcher drafted an expanded text for each recommendation, using logical operators (e.g. ‘AND’ and ‘OR’) to link descriptive statements to produce numerators and denominators (*n* = 1) • method not specified in detail (*n* = 15) • not reported (*n* = 3)
6. Intended use of performance measure	• quality improvement (*n* = 10) • quality of care delivered (*n* = 2) • monitoring compliance with guideline (*n* = 4) • implementation of care (*n* = 1) • clinical audit (*n* = 1) • pay for performance program (*n* = 1) • not specified (*n* = 8) • unclear (*n* = 1) • not reported (*n* = 2)
7. Practice test of performance measures	• planned (*n* = 18) • retrospective (n = 2) • implicit (*n* = 1) / ad hoc (*n* = 1) • not reported (*n* = 8)
8. Review and evaluation of performance measure	• plan for evaluation and updating (*n* = 3) • evaluation including criteria for retiring (*n* = 1) • mentioned, but not explained in detail (*n* = 2) • evaluation not reported, often because QI were developed but not yet implemented (*n* = 24)
9a. Composition of the panel	• monodisciplinary (*n* = 2) • multidisciplinary (*n* = 23) • panel composition not reported (*n* = 5)
9b. Composition of the panel: patient involvement	• yes (*n* = 10) • no (*n* = 17) • depends on guideline (*n* = 1) • not reported (*n* = 2)

^a^multiple criteria per methodological framework

#### Quality indicator development and GIN-McMaster guideline checklist (Table 2)

Most of the domains in guideline development had a corresponding domain in quality indicator development, but reporting was not optimal. The methods varied in many aspects, for example for group membership (domain 3) and the criteria for quality indicator selection (domain 5). None of the methods papers reported explicitly on establishing group processes, reporting and peer review, conflicts of interest considerations and updating of the quality indicators.

Nine of the 30 method articles described an approach based on one specific guideline [6, 11, 13–15, 21, 26, 32, 37]. While in the other articles, multiple guidelines and other sources were used in order to select potential quality indicators (domain 11).

#### Quality indicator development process (Table 3)

The overall approach observed in quality indicator development was that, based on the guideline(s) and other sources, a list of potential quality indicator was compiled (item 1 and 2 GIN reporting standards). The quality of the guidelines which were used as source for the quality indicator was appraised in 11 method articles, the Appraisal of Guidelines for Research and Evaluation (AGREE) tool was used in 8 of these and in four the criteria for appraisal was not fully specified (item 1b) [6, 7, 9, 14, 17, 23–25, 31, 36, 37]. This list with potential quality indicators served as input for a consensus approach, formal methods, often a modified RAND/UCLA (Delphi) approach, as well as informal methods were used to select the final set of quality indicator (item 3). In the included articles the criteria for selection of quality indicator varied, but could be grouped into relevance, evidence-based, feasibility and measurability (item 4). The potential for quality improvement and improving patient outcomes, scientific soundness and feasibility were most often mentioned as criteria for selecting quality indicator (item 4). How the criteria were defined and scored was not described in detail in most reports (item 5). Quality improvement was the most often mentioned reason for quality indicator development, but in 11 articles this was unclear or not reported (item 6). A practice test was planned for the majority of the methods (item 7). Evaluation of the quality indicator set was largely not reported (item 8). The panel composition was mostly multidisciplinary (item 8), there was no patient involvement in over half of the methods articles (item 9).

#### Linking guideline recommendations to quality indicator development

All but one method article started with describing the quality indicator development process and how the evidence reported in guidelines was used. One article described both development of recommendations and the set of quality indicators for those recommendations. It was unclear, however, how recommendations and quality indicators were linked [15]. None of the articles reported a framework in which quality indicator development was part of the question formulation for developing the guideline recommendations.

Seven of the 30 approaches linked guideline recommendations to quality indicator development, albeit in different ways and for different purposes [11, 14, 15, 21, 26, 32, 37]. Examples of the different purposes are to integrate quantitative measurements of quality and performance into the development cycle of existing and future therapeutics (via guidelines) [32], to derive structured quality indicator and auditing protocols from formalized specifications of guidelines used in decision support systems [26] and to measure quality of care, adherence to guideline recommendations, internal quality management for medical institutions and for benchmarking with other institutions [14, 21].

Three of the seven approaches with a linked approach used the level of evidence to select recommendations suitable for QI development [11, 14, 32]. The method articles from the American Thoracic Society (ATS) and the German Guidelines Program in Oncology (GGPO) report using the Grading of Recommendations Assessment, Development and Evaluation (GRADE) approach to develop recommendations and suggest that strong recommendations should be considered for translation into quality indicators [11, 14]. The third article mentioned level of evidence as an important characteristic of guidelines but was not explicit on how to use it in quality indicator development [32].

Three articles reported challenges for linkage. For Kahn and colleagues, rewording the recommendations to quality indicators and translating the quality indicators into measurable performance indicators with clearly defined numerators and denominators was challenging [11]. In two articles the challenges referred to the use of evidence, or more specifically, the lack thereof. Schleedoorn and colleagues reported that 11 of the 17 selected recommendations were good practice points (described as expert opinion), and six recommendations were derived from evidence described as Level A. According to the authors, this demonstrates the importance of expert opinion in daily practice [13]. Werbrouck et al. addressed this point as well, where authors remarked that very few process quality indicators in the final list had a high level of evidence. They stated that, “this is either due to the difficulty of providing a high level of evidence for some processes, such as pathology, or due to a real lack of clear evidence from randomized controlled trials for some clinical questions, such as the role of lymphadenectomy. The high mean scores attributed to these quality indicators by the expert’s panel clearly indicate their clinical value emphasizing that evidence should not be the only criterion to select quality indicator since it eliminates indicators deemed relevant by consensus.” [12]

## Discussion

### Summary of findings

We conducted an extension and update of a previous systematic review to identify approaches to the integrated development of guidelines and related quality indicator. We identified 30 articles describing these approaches, however, in general, these were not based on well-defined conceptual frameworks and lacked full integration of the two areas. Our key findings indicate a lack of coherence between the two fields and heterogeneity in methods. For example, the quality of the guidelines was not assessed in the majority of the articles. This suggests that although quality indicator development is often done on the basis of recommendations by reputable organizations, the suitability and quality of the recommendations may not coincide with the goals of quality indicators. There were no studies that evaluating the impact of guideline integrated quality indicator development on health outcomes. Almost 10 years ago, Kötter et al. came to the same conclusion that there continues to be a lack of impact evaluation of integrated frameworks [2].

The original review included 14 method articles and 32 topic articles; in the update (2010–2019) we found 17 new method articles and twice as many topic papers. This suggests that although quality indicator development is a topic of high interest, there is minimal methodological advancement and the connection with guideline development methods is very limited. The reason for the limited connection is not yet clear, and need to be investigated. However, the fact that guideline developers and quality improvement researchers work in silos is well recognized (ref Two Worlds paper). From this systematic review we have learned some lessons that will influence future practice and quality indicator development.

The key findings that will help with selecting elements of the approaches include the different ways evidence is used in the recommendation selection process for generating quality indicator. Some authors report using certainty in the evidence (or level of evidence) [5, 15, 22, 32–34], others find using evidence challenging and confuse expert opinion with an interpretation of the evidence [12, 13, 40], and only a few approaches use strength of recommendation [6, 11, 14]. This supports the distinction between certainty of evidence and strength of the recommendation, and guidance how to apply these concepts in quality indicator development.

### Strengths and limitations of this review

Strengths of this review include the systematic approach, the conceptual categorization of the findings according to established tools (GIN-McMaster Checklist and GIN Reporting standards for guideline-based performance measures) [3, 4], and the large amount of new information that we revealed. Potential limitations to the methods of this review are the restriction to three languages and the fact that methods papers are sometimes hard to track. We mitigated these limitations by searching for methods papers on the websites of relevant organisations, consulting experts and checking topic papers for references on the method that was used.

### Implications for practice

For developers in both the guidelines and the QA and quality indicator fields, our review has identified a few existing approaches that may be used to support guideline-based quality indicator development and avoid duplication of effort. In addition, our review highlights that better integration should be sought. For example, quality indicator should be thought of during the formulation of guideline questions to achieve better integration. Finally, our review highlights that those working in quality improvement should distinguish between expert opinion and evidence in the development of quality indicator [40].

### Implications for research

Our review has important implications for research that include the development of a well-defined conceptual framework and testing of that framework.

## Conclusions

Our systematic review of the literature resulted in 30 articles describing approaches for guideline-based QA and quality indicator development. In most approaches, guidelines were used as a source of evidence to inform the QI development. The criteria to select recommendations from the guidelines (e.g. level of evidence or strength of the recommendation) and to generate, select and assess quality indicators varied widely and not all quality indicator development criteria may be addressed in guideline development. We found approaches where guideline and quality indicator development were linked explicitly, but none of the articles reported a well-defined conceptual framework that properly integrated quality indicator in the guideline process. Research and conceptual development needs to be done in this area and we describe some of these advancements in our accompanying articles (reference to both).

## Supplementary information

**Additional file 1.** Appendix A. Search strategies. Appendix B. List of predefined organizations for manual search. Appendix C. Screening forms. Appendix D. Data abstraction forms.

## Data Availability

Not applicable.

## Abbreviations

EC: European Commission
ECICC: European Commission Initiative on Colorectal Cancer
GIN: Guidelines International Network
GRADE: Grading of Recommendations Assessment, Development and Evaluation
PROSPERO: Prospective Register of Systematic Reviews
QA: Quality Assurance
