# Supplementary material for: Approaches of integrating the development of guidelines and quality indicators: a systematic review
Source: BMC Health Serv Res. 2020 Sep 16;20:875. doi: 10.1186/s12913-020-05665-w (PMC7493171; doi:10.1186/s12913-020-05665-w)
Supplement: Supplementary file 1 — Additional file 1. Appendix A. Search strategies. Appendix B. List of predefined organizations for manual search. Appendix C. Screening forms. Appendix D. Data abstraction forms. [file 12913_2020_5665_MOESM1_ESM.docx]

**Appendices**

Appendix A. Search strategies

**Kötter search strategy^1^**

#### Medline search algorithm

| ***Quality indicators*** | ***Clinical guidelines*** | ***Development*** |
| --- | --- | --- |
| 1. quality indicator$.tw | 12. guideline$.tw | 31. develop$.tw |
| 2. quality criterion$.tw | 13. practice guideline/ | 32. and\11,30,31 |
| 3. quality measure$.tw | 14. practice guideline$.tw |  |
| 4. performance indicator$.tw | 15. clinical practice guideline$.tw |  |
| 5. performance measure$.tw | 16. recommendation$.tw |  |
| 6. outcome measure$.tw | 17. guidance$.tw |  |
| 7. outcome indicator$.tw | 18. directive$.tw |  |
| 8. audit.tw | 19. health service$ research.tw |  |
| 9. outcome assessment.tw | 20. evidence based medicine.tw |  |
| 10. process assessment.tw | 21. quality assessment.tw |  |
| 11. or\1-10 | 22. quality assurance.tw |  |
|  | 23. consensus technique.tw |  |
|  | 24. delphi technique.tw |  |
|  | 25. RAND.tw |  |
|  | 26. UCLA.tw |  |
|  | 27. RAM.tw |  |
|  | 28. RAND appropriateness method.tw |  |
|  | 29. consensus development/ |  |
|  | 30. or\12-29 |  |

**UPDATE:**

**Database: OVID Medline Epub Ahead of Print, In-Process & Other Non-Indexed Citations, Ovid MEDLINE(R) Daily and Ovid MEDLINE(R) 1946 to Present**

Search Strategy:

1 quality indicator$.tw. (6531)

2 quality criterion$.tw. (290)

3 quality measure$.tw. (6184)

4 performance indicator$.tw. (2916)

5 performance measure$.tw. (9507)

6 outcome measure$.tw. (195834)

7 outcome indicator$.tw. (1365)

8 audit.tw. (29931)

9 outcome assessment.tw. (3254)

10 process assessment.tw. (171)

11 1 or 2 or 3 or 4 or 5 or 6 or 7 or 8 or 9 or 10 (250158)

12 exp practice guideline/ (23922)

13 guideline$.tw. (284968)

14 practice guideline$.tw. (21094)

15 consensus development/ (8824)

16 clinical practice guideline$.tw. (11646)

17 recommendation$.tw. (213343)

18 guidance$.tw. (91570)

19 directive$.tw. (14202)

20 health service$ research.tw. (3240)

21 evidence based medicine.tw. (11794)

22 quality assurance.tw. (22580)

23 consensus technique.tw. (75)

24 delphi technique.tw. (1401)

25 RAND.tw. (2742)

26 UCLA.tw. (4425)

27 RAM.tw. (5374)

28 RAND appropriateness method.tw. (72)

29 quality assessment.tw. (14293)

30 or/12-29 (614831)

31 develop$.tw. (3753758)

32 11 and 30 and 31 (7329)

33 limit 32 to yr="2010 -Current" (4306)

**Database: Embase <1974 to 2018 May 11>**

Search Strategy:

1 quality indicator$.tw. (9805)

2 quality criterion$.tw. (363)

3 quality measure$.tw. (8632)

4 performance indicator$.tw. (4198)

5 performance measure$.tw. (12157)

6 outcome measure$.tw. (246459)

7 outcome indicator$.tw. (1789)

8 audit.tw. (60661)

9 outcome assessment.tw. (4342)

10 process assessment.tw. (210)

11 1 or 2 or 3 or 4 or 5 or 6 or 7 or 8 or 9 or 10 (340232)

12 exp practice guideline/ (452218)

13 guideline$.tw. (437256)

14 practice guideline$.tw. (28599)

15 consensus development/ (22834)

16 clinical practice guideline$.tw. (15638)

17 recommendation$.tw. (292263)

18 guidance$.tw. (130807)

19 directive$.tw. (20018)

20 health service$ research.tw. (3866)

21 evidence based medicine.tw. (15102)

22 quality assurance.tw. (32468)

23 quality assessment.tw. (18514)

24 consensus technique.tw. (88)

25 delphi technique.tw. (1699)

26 RAND.tw. (3589)

27 UCLA.tw. (6172)

28 RAM.tw. (6560)

29 RAND appropriateness method.tw. (108)

30 or/12-29 (1119121)

31 develop$.tw. (4752110)

32 11 and 30 and 31 (14115)

33 limit 32 to yr="2010 -Current" (9814)

**Interface - EBSCOhost Research Databases | Search Screen - Advanced Search**

**Database – CINAHL**

**# Query Limiters/Expanders Results**

S5 S1 AND S2 AND S3 Limiters - Published Date: 20100101-20180131 3,820

S4 S1 AND S2 AND S3 7,664

S3 TX quality indicator* OR TX quality criterion* OR TX quality measure* OR TX performance indicator* OR TX performance measure* OR TX outcome measure* OR TX outcome indicator* OR TX audit* OR TX outcome assessment OR TX process assessment 121,369

S2 TX develop* 546,984

S1 TX guideline* OR TX practice guideline* OR TX guideline* OR MH consensus development OR TX clinical practice guideline* OR TX recommendation* OR TX guidance* OR TX directive* OR TX health service* research OR TX evidence based medicine OR TX quality assurance OR TX consensus technique OR TX delphi technique OR TX RAND OR TX UCLA OR TX RAM OR TX RAND appropriateness method 251,797

Appendix B. List of predefined organizations for manual search

#### Websites <add urls>

| ***Institution*** | ***URL*** |
| --- | --- |
| **Kötter** |  |
| American Academy of Otolaryngology – Head and Neck Surgery | http://www.entnet.org/ |
| Australian Council on Healthcare Standards | http://www.achs.org.au/ |
| AGREE  Collaboration | http://www.agreetrust.org/ |
| American Heart Association | http://www.heart.org/ |
| Agency for Healthcare Research and Quality | http://www.qualitymeasures.ahrq.gov/ |
| American Medical Association | http://www.ama-assn.org/ |
| Institute for Applied Quality Improvement and Research in Health Care (AQUA-Institute) | http://www.sqg.de/startseite/index-en.html |
| American Society of Clinical Oncology | http://www.asco.org/ |
| Arbeitsgemeinschaft der wissenschaftlichen medizinischen Fachgesellschaften (AWMF) | http://www.awmf.org/ |
| Agency for Quality in Medicine | http://www.aezq.de/ |
| BQS Institut für Qualität und Patientensicherheit | http://www.bqs-institut.de/ |
| Canadian Cardiovascular Outcomes Research Team | http://www.ccort.ca/ |
| German College of General Practitioners and Family Physicians | http://www.degam.de/ |
| EQUAM-Foundation | http://www.equam.org/ |
| Guidelines International Network | http://www.g-i-n.net/ |
| Initiative for Maternal Mortality Programme Assessment (Impact) | http://www.immpact-international.org/ |
| IQ Healthcare | http://www.iqhealthcare.nl/ |
| The Joint Commission | http://www.jointcommission.org/ |
| The National Association of Statutory Health Insurance Physicians | http://www.kbv.de/ |
| National Committee for Quality Assurance | http://www.ncqa.org/ |
| National Institute for Clinical Excellence | http://www.nice.org.uk/ |
| National Primary Care Research and Development Council | http://www.medicine.manchester.ac.uk/primarycare/npcrdc-archive/index.cfm.htm |
| New Zealand Guidelines Group | http://www.nzgg.org.nz/ |
| Organisation for Economic Cooperation and Development | http://www.oecd.org/ |
| RAND Corporation | http://www.rand.org/ |
| Royal College of General Practitioners | http://www.rcgp.org.uk/ |
| Royal College of Physicians | http://www.rcplondon.ac.uk/ |
| Scottish Intercollegiate Guidelines Network | http://www.sign.ac.uk/ |
| **Additional websites** |  |
| National Health and Medical Research Council (NHMRC) |  |
| U.S. Preventive Services Task Force (USPSTF) |  |
| Canadian Task Force on Preventive Health Care (CTFPHC) |  |
| Public Health Agency of Canada (PHAC) |  |
| American College of Physicians (ACP) |  |
| World Health Organisation (WHO) |  |
| European Commission Joint Research Centre (EC-JRC) |  |
| [The German Hospital Federation](http://www.dkgev.de/dkg.php/cat/256/title/The_German_Hospital_Federation) (DKG) |  |
| Arbeitsgemeinschaft der Wissenschaftlichen Medizinischen Fachgesellschaften (AWMF) | [www.awmf-online.de](http://www.awmf-online.de) |
| International Society of Quality in Health Care (ISQUA) |  |
| Duodecim |  |
| Canadian Colorectal Cancer screening guidelines and quality indicators |  |

Appendix C. Screening forms

**A systematic review to identify and evaluate the effects of approaches to integrate QA/QI/PI scheme and guideline development**

Title and abstract screening guide

1. Does the report describe development of QA/QI/PI using evidence-based guideline recommendations? [see definitions of QA/QI/PI below – all aspects of quality of care are eligible]

Tips:

- Include reports about QI development using (modified) Delphi/RAND methodology and no specific mentioning of guideline recommendations in abstract as this approach often uses guideline recommendations;
- Include reports using terms like ‘evidence-based’, ‘scientific literature’, ‘medical literature’, ‘evidence from literature’, instead of specific mentioning guidelines or recommendations;
- Result of the studies we are interested in is a set of QI/PI or QA (or set of health care recommendations based on quality aspects), or description of the method without application to health care topic;
- Exclude reports where indicators are used to measure quality of care, in patients or health systems – we are only interested in the development method
- Exclude reports on the development of a specific guideline without any mentioning of QI development in the abstract
- Exclude reports on assessing or evaluating measurement properties of outcome measurement instruments (e.g. COSMIN, PRO)
- No => Exclude
- Yes or Uncertain => Next question

1. Type of report:

- Manual/SOP/handbook/guidance document that describes an applied or hypothetical approach to integrate the development of recommendations and QA/QI/PI => Include
- Report that describes an application of an integrated approach (i.e. guideline or QI set on a specific topic) => Include
- Report that evaluates integrated approach (comparative or non-comparative) on health or process outcomes => Include
- Review or methods paper addressing integrated approaches (e.g. guideline-based QA/QI/PI development) => Include
- Other => Exclude
- Uncertain => Include

**Notes:**

- We will not use any language restrictions
- If uncertain about a certain abstract then include it
- If you come across duplicate studies indicate that it was excluded as it was duplicate even if the study is excluded (to keep track of the duplicate numbers)

**A systematic review to identify and evaluate the effects of approaches to integrate QI/QA scheme and guideline development**

Full text screening guide

Study ID:

First author:

Year:

Screener initials:

1. Does the report describe development of evidence-based recommendations **and** development of QA/QI/PI [see definitions]?

- No => Exclude
- Yes or Uncertain => Next question

1. Is the development of recommendations and QA/QI/PI part of an intentional integrated approach?
   Intentional integrated means that recommendations are used to develop (part) of the QA/QI/PI, or that the recommendations are based on/follow from the QA/QI/PI development.
   The approach could be a parallel or serial.

- No => Exclude
- Yes or Uncertain => Next question

1. Does the report provide a description of the methodology used to link recommendations and QA/QI/PI?

- No => Exclude
- Yes or Uncertain => Next question

1. Is the description of the development detailed enough to identify at least the following components for one or more recommendations in the report: (PICO) question generation, summarizing evidence, considering additional information, developing recommendations and development of QA/QI/PI?

- No => Exclude
- Yes or Uncertain => Next question

1. Type of report:

- Manual/SOP/handbook/guidance document that describes an applied or hypothetical approach to integrate the development of recommendations and QA/QI/PI => Include
- Report that describes an application of an integrated approach (i.e. guideline on a specific topic) => Include
- Report that evaluates integrated approach (comparative or non-comparative) on health or process outcomes => Include
- Review or methods paper addressing integrated approaches (e.g. guideline-based QA/QI/PI development) => Include
- Other => Exclude
- Uncertain => Include

**Reasons for exclusion:**

- Report not about recommendations and QA/QI/PI
- Not intentional integrated approach
- Methodology of integration not described
- Description not detailed enough
- Duplication of prior report (use the most complete)
- Not a manual, application or evaluation study

**Notes:**

- We will not use any language restrictions
- If uncertain about a certain abstract then include it
- If you come across duplicate studies indicate that it was excluded as it was duplicate even if the study is excluded (to keep track of the duplicate numbers)

Appendix D. Data abstraction forms

**Kötter review data abstraction form**

#### Data extraction form

| *Item* | *Extracted information* | | | | | | | | | | |
| --- | --- | --- | --- | --- | --- | --- | --- | --- | --- | --- | --- |
| Design | Method paper | | | Topic paper | | | | Method + topic paper | | | |
| Publication type | Not mentioned | | | Mentioned in full text | | | | Mentioned on title page | | | |
| Duration of the study | Not mentioned | | | | | Mentioned | | | | | |
| Funding | Not transparent | Public funding | | | | Private funding | | | Both public / private | | |
| Topic selection | No criteria reported | | | | | Criteria reported | | | | | |
| Target population / setting | Not reported | | | Reported | | | | Method paper | | | |
| Panel method | Modified RAND / UCLA | Other | | | | Unclear | | | No panel method | | |
| Selection criteria for panel members | Not reported | Reported | | | | Unclear | | | No panel method | | |
| Panel members | Not mentioned | | | Mentioned | | | | No panel method or method paper | | | |
| Selection criteria for persons, who extracted recommendations | Not reported | | | | | Reported | | | | | |
| Extracting persons | Not mentioned | Mentioned | | | | Unclear | | | Method paper | | |
| Development of QI from... | One guideline | | | More than one guideline | | | | Guideline(s) and other sources | | | |
| Method for guideline selection | Not mentioned | | | Mentioned | | | | Unclear | | | |
| Critical appraisal of guidelines | Not conducted | | | Conducted | | | | Unclear | | | |
| Selected guidelines | Not mentioned | | | Mentioned | | | | Method paper | | | |
| Extracted recommendations | All | | | Selection | | | | Unclear | | | |
| Criteria for selection of recommendations | Not mentioned | | Mentioned | | | | Unclear | | | | No selection |
| Potential indicators | Not listed | | | | Listed | | | | | Method paper | |
| Selected indicators | Not listed | | | | Listed | | | | | Method paper | |
| Sources of the single indicators | Not mentioned | | Partially mentioned | | | | Always mentioned | | | | Method paper |
| LoE of underlying recommendations | Not mentioned | | | | Mentioned | | | | | Unclear | |
| Assessment of QI using explicit criteria | Not mentioned | | | | Mentioned | | | | | Unclear | |
| Practice test | Not mentioned | | Included | | | | Planned | | | | Proposed |
| Implementation strategy | Not mentioned | | | | | | Mentioned | | | | |
| Patient participation | No participation | | | | Guideline selection / extraction | | | | | QI selection | |

QI = quality indicator; LoE = level of evidence.
